# Borderline Personality Disorder (BPD): In the Midst of Vulnerability, Chaos, and Awe

**DOI:** 10.3390/brainsci8110201

**Published:** 2018-11-18

**Authors:** Filiz Kulacaoglu, Samet Kose

**Affiliations:** 1 Department of Psychiatry, Cerkezkoy State Hospital, Tekirdag 59500, Turkey; fkulaca@gmail.com; 2 Department of Psychology, Hasan Kalyoncu University, Gaziantep 27000, Turkey; 3 University of Texas Medical School of Houston, Houston, TX 77065, USA; 4 Center for Neurobehavioral Research on Addictions, Houston, TX 77054, USA

**Keywords:** borderline personality disorder, etiology, comorbidity, treatment

## Abstract

Borderline personality disorder (BPD) is a chronic psychiatric disorder characterized by pervasive affective instability, self-image disturbances, impulsivity, marked suicidality, and unstable interpersonal relationships as the core dimensions of psychopathology underlying the disorder. Across a wide range of situations, BPD causes significant impairments. Patients with BPD suffer considerable morbidity and mortality compared with other populations. Although BPD is more widely studied than any other personality disorder, it is not understood sufficiently. This paper briefly reviews the recent evidence on the prevalence, etiology, comorbidity, and treatment approaches of borderline personality disorder (BPD) by examining published studies, and aims to offer a more coherent framework for the understanding and management of borderline personality disorder.

## 1. Introduction

Borderline personality disorder (BPD) is a chronic psychiatric disorder characterized by pervasive patterns of affective instability, self-image disturbances, instability of interpersonal relationships, marked impulsivity, and suicidal behavior (suicidal ideation and attempt) causing significant impairment and distress in individual’s life [1]. Patients with BPD suffer considerable morbidity which complicates medical care compared to other individuals. BPD was initially defined in 1978 followed up with the publication of the Diagnostic and Statistical Manual of Mental Disorders, Third Edition (DSM-III) in 1980 [2] and International Classification of Diseases (ICD-10) [3] 10 years later. It has become a diagnosis based on the systematic identification of clinical features and identified as emotionally unstable personality disorder [2]. Both the DSM-5 [4] and ICD-10 [3] highlighted the affective instability as an essential criterion for BPD. Individuals with BPD have an underlying vulnerability to emotional hyperarousal states due to abnormalities in neurobiological systems sub-serving emotional regulation and stress responsibility. They also have an underlying vulnerability to social and interpersonal stressors due to abnormalities in neurobiological systems mediating social cognition, attachment, and social reward. Under stressful conditions, BPD patients are unable to regulate their emotions and quickly return to their baseline emotional states.

Since BPD is associated with receiving clinical attention and causes psychosocial impairments, it is more widely studied than other personality disorders [5,6]. In this brief review, we aim to elucidate epidemiology, pathogenesis, clinical features, comorbidity, and treatment approaches to BPD by critically examining published studies.

## 2. Epidemiology

The lifetime prevalence of BPD is approximately 5.9% and the point prevalence of BPD is 1.6% [6,7]. Although the prevalence of BPD is not higher than other personality disorders in the general population, BPD has a high prevalence in treatment settings; BPD was present in 6.4% of primary care visits, 9.3% of psychiatric outpatients and 20% of psychiatric inpatients according to the studies in clinical settings [4,8,9]. However, the ratio of females to males with the disorder is also greater in the clinical population. The ratio is 3:1 in clinical settings cited in the DSM-5 [4]. In contrast to the clinical setting ratio, in two epidemiologic surveys of United States general population, the lifetime prevalence of BPD was found to be similar in males and females [6,7]. This result can be interpreted as women with BPD are more likely to seek treatment than men. About 80% of patients who receive treatment for BPD were reported to be women.

## 3. Pathogenesis

The cause of BPD is not known and it is suggested that BPD is the product of an interaction between genetic, neurobiological, and psychosocial influences that affect brain development [10].

Although studies are rare and different values have been reported, there is at least moderate evidence for the genetic transmission and heritability of BPD. According to two studies, the concordance rate for BPD was found to be higher in monozygotic twins compared with dizygotic twins (36 and 35% versus 19 and 7%) [11,12]. However, a third twin study reported that a common genetic influence has little contribution to the development of BPD compared to environmental influences (42% versus 58%) [13]. In sum, constitutional predisposition to emotional dysregulation with a non-supporting environment leads to the development of BPD [14]. Future studies are needed to focus on interactions of specific endophenotype and environmental factors.

According to neurobiological research data, it has been suggested that neuropeptide functions may predispose to interpersonal problems of BPD patients [15]. The hypothalamic pituitary adrenal (HPA) axis dysfunction has a central role in the development of BPD. Increased levels of stress hormones, such as basal cortisol, and reduced feedback sensitivity were reported in BPD patients [16]. However, maladaptive behaviors of self–others and relationships with others are believed to be modulated by the oxytocinergic system [17]. Increased HPA activity and decreased peripheral oxytocin levels are correlated with a history of early life maltreatment and insecure attachment in patients with BPD [18]. Moreover, few studies also reported increased testosterone levels in female and male patients with BPD [16].

Neuroimaging studies that have compared BPD patients with healthy controls have reported bilateral reductions in the hippocampus, amygdala, and medial temporal lobe [19,20]. The neurobiology of BPD can be conceptualized as abnormalities in the top-down control, provided by the orbitofrontal cortex and the anterior cingulate cortex, and the bottom-up control drives generated in the limbic system such as amygdala, hippocampus, and insular cortex. Top-down control provides cognitive control areas and bottom-up control provides salience detection [21]. In this circuitry, serotonin regulates the prefrontal regions by acting on 5-HT2 receptors in a different role [22]. Impulsive traits, a major component of BPD, are associated with deficits in central serotonergic functioning. More specifically, increased 5-HT2A receptors and decreased 5-HT2C receptors are related with impulsivity [21]. Impulsivity is a core feature of BPD and it is related with reward and control circuits and deficient behavioral inhibition in prefrontal areas [23]. However, left amygdala hyperactivity was found in unmedicated patients with acute BPD. This feature is consistent with negative environmental stimuli [24]. Intense and variable emotions of BPD patients are related with amygdala hyperactivity. The role of the amygdala also reflects maladaptive top-down processes in evaluating negative environmental stimuli [25]. However, an enlarged hypothalamus and dysregulated HPA axis, and a reduced volume of the amygdala and hippocampus are found in patients with a history of early trauma and posttraumatic stress disorder (PTSD) [26,27,28]. In addition, the finding of reductions in gray matter volume of amygdala in older BPD patients has been interpreted as reflecting a reversible progressive pathology [29]. Emotional regulation difficulties of BPD patients are related with insufficient capacity of cognitive processes of prefrontal cortex (PFC) activity [30]. Koenigsberg et al. reported hypoactivity in orbitofrontal cortex, ventrolateral cortex, and dorsal anterior cingulate cortex (ACC) in BPD patients compared with healthy individuals [31]. This result is related with maladaptive affective regulation in BPD patients. However, lower prefronto-limbic connectivity within the affect regulation circuitry was reported to be normalized after successful psychotherapy [32]. In sum, numerous studies that have compared BPD patients with healthy controls reported a serotonergic dysfunction and reductions in amygdala, hippocampus, and medial temporal lobe volumes [19,20]. However, since these studies enrolled adult BPD patients, it is not clear that these neurobiological defects are the sequelae or etiologic causes of the disorder.

Life experiences are also known to be associated with the development of BPD [33]. Childhood trauma is the most significant risk factor for development of BPD [34]. Since childhood trauma is not always present in BPD, and individuals who had trauma do not always necessarily develop BPD, this relationship between childhood trauma and BPD is not clear. It can be interpreted that childhood trauma is not a mandatory precondition for the development of BPD. Childhood trauma in BPD patients can take many forms in prospective studies including sexual abuse, physical abuse and neglect, verbal abuse, and early parental separation or loss [35]. According to a prospective study with 500 individuals, more physically abused and/or neglected children met the criteria of BPD as adults. Interestingly, sexual abuse history is not found as a risk factor for BPD. However, having a parent with alcohol or substance use problems, having a diagnosis of drug abuse, major depressive disorder, and post-traumatic stress disorder have all been associated with the development of BPD but are also non-specific factors [36]. Another prospective, longitudinal study with 639 children reported that childhood abuse/neglect was significantly associated with BPD in adulthood [37]. Meta-analyses have also found that only small effect sizes for the relationship between development of BPD and childhood maltreatment [38,39]. As with most psychiatric disorders, no single factor can explain the development of the disorder, multiple factors can help in explaining the development of BPD. Although, there were studies that reported that childhood trauma did not play a significant role in the development of BPD, it still remains an important risk factor for BPD and more studies are needed to elucidate this relationship.

## 4. Clinical Features and Comorbidities

BPD is a psychiatric disorder, which was initially thought to emerge during adolescence and continue into adulthood [40]. It has also been stated that a diagnosis starts from adolescents in DSM-5 [4]. According to DSM-5 Section II, the diagnostic criteria of BPD are divided into four dimensions: (a) Interpersonal instability dimension, which has the features of fear of abandonment and intense unstable relationships; (b) cognitive and/or self-disturbance, which consists of paranoid ideations, dissociative symptoms, and identity disturbances; (c) affective and emotional dysregulation; and (d) behavioral dysregulation dimension, which has impulsivity and suicidal behavior [4].

Affective instability has been shown to be the most specific, sensitive criteria for BPD [41]. Patients with BPD are emotionally labile, react strongly, and express dysphoric emotions such as depression, anxiety, and irritable mood [42]. However, a study that examined the associations of age with affective instability of BPD patients showed an inverse relationship between age and affective instability in patients with BPD [43]. Patients with BPD have unstable and conflicted relationships. They tend to view others as all good and bad which is labeled as ‘splitting’. They can easily become dependent on others but they can also have dramatic shifts in their feelings toward others. Cognitive dysfunction in BPD patients has also been shown in a meta-analysis, where BPD patients scored poorer on tests of attention, cognitive flexibility, planning, learning, and memory [44].

Impulsive behavior is a core feature of BPD and might take many forms. Substance abuse, impulsive spending, binge eating, reckless driving, and self-damaging behavior are very common and put the patient at risk of harm [45]. Previous studies suggested that impulsivity, emotional dysregulation, and self-harm behaviors during childhood are predictive features of BPD [46].

Suicidal attempts and ideations are common manifestations of BPD and are one of the diagnostic criteria of DSM-5 [4]. In retrospective studies, the rate of suicide is found to be 8%–12% in BPD individuals [47]. Suicidal tendency is most common at age 20 [48], and completed suicide attempts are more common after the age of 30 years in patients with BPD [48]. Patients may also engage in suicidal behaviors, such as cutting themselves. These behaviors, ideation or acts might be conceptualized as non-suicidal self-injury [49]. Since non-suicidal acts and suicide attempts are so common in BPD patients, it is quite difficult to assess the current risk of a patient’s suicidal intent. Patients who have attempted suicide more than once have an increased risk for completed suicide. According to prospective studies, the predictors of suicide in patients with BPD were reported as co-occurring symptoms of dissociation, affective reactivity, self-harm, depression comorbidity, family history of suicide, and history of childhood abuse [50,51]. According to a recent study, which examined gender differences and similarities in aggression, psychiatric comorbidity, and suicidal behavior in patients with BPD, men with BPD were found more aggressive, impulsive and more impaired than women with BPD. Men with BPD were found at higher risk of dying due to a suicide attempt compared to women with BPD [52].

Comorbid psychiatric disorders are common in patients with BPD [53]. According to an epidemiologic survey, 85% of BPD patients have at least one comorbid psychiatric disorder [6]. Mood disorders, especially depressive disorder, bipolar disorder, anxiety disorder, posttraumatic stress disorder (PTSD), substance use disorder, or other personality disorder and neurodevelopmental disorder such as attention-deficit/hyperactivity disorder (ADHD), might be present in patients with BPD [54]. According to several large patient samples, the rate of lifetime depression comorbidity ranges from 71% to 83%, and anxiety disorder comorbidity is as high as 88% in patients with BPD [55,56]. More recently in a genome-association study by Witt et al., genetic overlap has been found between BPD and bipolar disorder, major depressive disorder, and schizophrenia [57]. Their findings supported the role of genetic factors having a role in the development of BPD.

### 4.1. Borderline Personality Disorder and Bipolar Disorder

Borderline personality disorder (BPD) and bipolar disorder can co-occur in 10%–20% of cases and since symptomatology of these disorders is very similar, many patients with BPD have been mistakenly diagnosed with bipolar disorder [58]. It has also been suggested that BPD should be conceptualized as a part of the bipolar spectrum [59,60]. Smith et al. reported that a significant percentage of patients with BPD were in the bipolar spectrum [61], while Paris et al. reported that no empirical evidence supported BPD’s link to the bipolar spectrum [62]. By reviewing neuroimaging studies, Sripada and Silk reported that there were both overlap and differences in certain brain regions between BPD and bipolar disorder individuals [63]. A higher but not significant prevalence of BPD in patients with bipolar II disorder was reported [56] and Vieta et al. reported that BPD was diagnosed twice as frequently in patients with bipolar II disorder and bipolar I disorder [64]. Zimmerman et al. reported that patients with major depressive disorder (MDD) and BPD had excess psychosocial morbidity compared to MDD patients without BPD, and that BPD was the third most frequent diagnosis in patients with bipolar disorder after obsessive-compulsive disorder and histrionic personality disorder, respectively [65]. In sum, these results can be interpreted as each disorder is diagnosed in the absence of the other and these findings challenge the notion that BPD can be conceptualized as the part of the bipolar spectrum [66].

### 4.2. Borderline Personality Disorder and Early Trauma History

Trauma history is a central feature of both PTSD and BPD. The neurobiological impairments associated with the development of BPD can be conceptualized as the predisposing factor for BPD. Both environmental and neurobiological factors contribute to the development of BPD. Genetic predisposition becomes activated during environmental experiences of trauma history. It has been reported that trauma and neglect might exacerbate both biological and behavioral tendencies [67]. However, sufficient maternal care may buffer these vulnerabilities. These results might explain why some emotionally dysregulated individuals do not develop BPD despite their genetic tendencies. There is also evidence for a strong association between traumatic events and dissociative symptoms in BPD [68]. According to retrospective studies, borderline patients have high rates of childhood abuse and dissociation [69]. Depersonalization/derealization are core symptoms of BPD and dissociation can be a prominent feature in some individuals with BPD. Research in the dissociative subtype of PTSD and depersonalization suggested that dissociation might be a form of emotional over-modulation, promoting trauma-related stressful emotions [70]. Dissociation severity was predicted by the childhood traumas such as inconsistent caretaking, sexual abuse, adult rape, emotional neglect [71].

### 4.3. Borderline Personality Disorder and ADHD

The comorbidity of ADHD has been reported in 20% of BPD patients in several studies [72]. Since impulsivity is considered to be a central feature of BPD and ADHD, impulsivity has been examined as part of adult ADHD symptomatology in BPD patients. According to Philipsen et al., ADHD should be considered as a potential risk factor in patients with BPD with impulsivity [73]. In a recent study that has examined the association between impulsivity and ADHD in BPD patients, we reported higher comorbidity of ADHD in BPD group, and motor impulsiveness has been shown as a potential predictor of ADHD symptoms in BPD group [74]. In terms of the relationship between BPD, ADHD, and impulsivity, BPD-ADHD has been considered a severe, more impulsive and homogeneous subtype of BPD [75].

In sum, since BPD has been associated with chronic course of other psychiatric disorders, clinicians should carefully evaluate comorbid psychiatric conditions in patients with BPD in order to plan appropriate treatments.

## 5. Treatment

Since patients with BPD suffer considerable morbidity and mortality, BPD causes a therapeutic challenge for clinicians. First-line treatment for BPD is psychotherapy [76]. However, symptom targeted medications have also been found effective [77].

The psychotherapies that have been adapted to treat patients with BPD are; Dialectical behavior therapy (DBT), Mentalization-based therapy, Transference-focused therapy, Cognitive-behavioral therapy (CBT), and Schema-focused therapy [78]. These therapies provide active and focused interventions that emphasize current functioning and relationships. These therapy modalities also provide; (a) a structured manual that supports the therapist and provides recommendations for common clinical problems; (b) they are structured so that they encourage increased activity, proactivity, and self-agency for the patients; (c) focus on emotional processing, particularly on creating robust connections between acts and feelings; (d) increased cognitive coherence in relation to subjective experience in the early phase of treatment by including a model of pathology that is carefully explained to the patient, and encouraging an active stance by the therapist, which invariably includes an explicit intent to validate and demonstrate empathy and generate strong attachment relationships to create a foundation of alliance. Psychoeducation is also an important part of BPD treatment. It includes informing patients and families about the disorder, signs and the symptoms of the disorder, and also possible causes and treatment options [79]. According to a 2017 systematic review and meta-analyses of 33 clinical trials with 2256 participants that examined the efficacy of psychotherapies for BPD, DBT and psychodynamic approaches were found more effective compared to other psychotherapy modalities [80]. An earlier 2012 systematic review and meta-analyses had reported DBT, mentalization-based, transference-focuses and schema-focused therapies are effective for BPD treatment. But the results for CBT have mixed results [81]. DBT is a well-studied form of CBT that puts emphasis on impulsive behavior and affective instability, and aims to regulate emotional lability using group or individual sessions. According to a clinical study that consisted of 101 women with BPD and self-injurious behavior who received DBT over a two-year period, fewer patients treated with DBT attempted suicide and required psychiatric hospitalization (23% versus 46%) compared with patients received community treatment [80]. DBT focuses on improving coping skills, self-destructive behavior and acting out. Mentalization-based and transference-focused therapies are primarily psychodynamic therapies. Mentalization therapy also includes cognitive techniques. For example, the patient is supported to observe her mind and create alternative perspectives of her thoughts to others. Transference-focused therapy includes confrontation, exploration and transference interpretations for the relationships of the BPD patients with other individuals. Schema-focused therapy is a form of CBT that includes skills training. Family education can be used adjunct to other therapies for BPD treatment [78].

According to the literature, the pharmacological treatment for BPD is limited. It is suggested that the patient with BPD who continues to experience severe, impairing symptoms (for example affective dysregulation, impulsive-behavioral dyscontrol, perceptual symptoms) despite receiving psychotherapy, should receive symptom-focused, adjunctive medication treatment [42]. According to the clinical surveys and meta-analyses, low-dose antipsychotic drugs are more effective for cognitive and perceptual symptoms such as dissociation, paranoid ideation, and hallucinations compared with antidepressants or mood stabilizers. Mood stabilizers are found to be more effective for impulsivity, aggression, and behavior control in BPD [77]. Mood stabilizers in the meta-analyses were lamotrigine, topiramate, valproate, and lithium. Lithium is also found to be effective in preventing suicide in BPD patients as reported by a retrospective study. But lithium has a limited usage due to significant side effects [82]. However, according to preliminary evidence, omega-3 fatty acids are suggested as adjunct to primary medication treatment, with mood stabilizers to prevent recurrent self-harms [83]. Meta-analyses have also found that mood stabilizers and low-dose antipsychotics are more effective for affective dysregulations in BPD compared to antidepressants [77].

Since BPD has a high rate of psychiatric comorbidity, clinicians should be aware of co-occurring mood and anxiety disorders, and substance use disorder for treating patients with BPD. For mood and anxiety disorders, clinicians should be careful to prescribe higher doses of antidepressant drugs for treating subthreshold symptoms. Thus, clinicians should focus on BPD treatment and effective treatment should be organized for comorbid psychiatric situations for patients with BPD. However, when it comes to substance use disorder, bipolar disorder comorbidity and treatment of the substance use disorder should take precedence over BPD for safety [84]. There is no evidence supporting the use of polypharmacy in personality disorder. According to the US FDA, no medication is approved and no class of psychoactive medication is dramatically effective [85]. However, The National Institute for Health and Care Excellence (NICE) guidelines have reported that psychotropic medication should not be used to treat the BPD and may be prescribed for symptoms of co-occurring disorders for a short period of time [86]. In sum, treatment of BPD is multimodal. Psychotherapy is the first line treatment and adjunctive, symptom focused pharmacotherapy is essential. Comorbid psychiatric disorders should be assessed. A positive therapeutic alliance with patient and family, as well as psychoeducation about the nature of the disorder, are useful to maintain the treatment.

## 6. Conclusions

Borderline personality disorder (BPD) is a psychiatric disorder that causes significant impairment with a high prevalence occurring in adolescence and early adulthood. The disorder is associated with more clinical attention than other personality disorders and has a risk for higher suicidality. The etiology is still unknown. According to the literature, a combination of genetic factors, neurobiological abnormalities and childhood trauma history can cause development of BPD. BPD can be conceptualized as a chronic and persistent disorder. However, according to prospective studies, higher rates of remission and recurrence have been reported. There is still a lack of information on which factors lead to the development of BPD. Further studies are necessary to understand the pathology of BPD and to help reach the best choices of treatment for clinicians. Most psychotropic medications were found to be effective in treatment of symptoms of affective dysregulation and impulsive aggression, which have been the core dimensions of underlying psychopathology. Polypharmacy practice is not evidence-based and is unnecessary in the management of patients with BPD.
